# Supplementary figures and images for: Development of an observational exposure human biomonitoring study to assess Canadian children’s DEET exposure during protective use
Source: PLoS One. 2022 Aug 4;17(8):e0268341. doi: 10.1371/journal.pone.0268341 (PMC9352095; doi:10.1371/journal.pone.0268341)

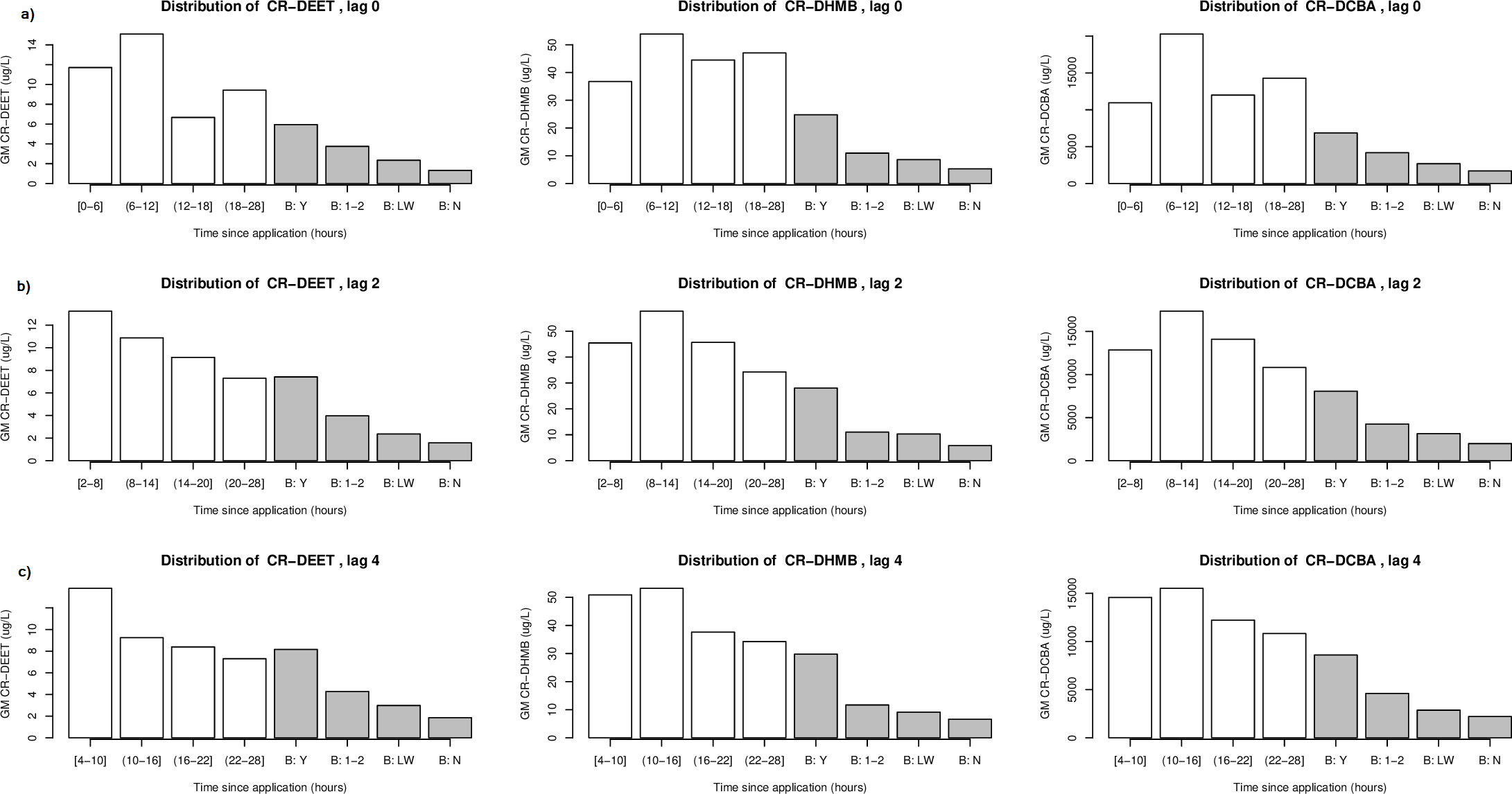

Supplement: S1 Fig — (a) distribution with a lag of 0 hours, (b) distribution with a lag of 2 hours, (c) distribution with a lag of 4 hours. (B:Y—Baseline group: Yesterday; B:1–2—Baseline group: 1–2 days ago; B:LW—Baseline group: Last week; B:N—Baseline group: Never). (TIF) [file pone.0268341.s001.tif]
