# Supplementary material for: Development of an observational exposure human biomonitoring study to assess Canadian children’s DEET exposure during protective use
Source: PLoS One. 2022 Aug 4;17(8):e0268341. doi: 10.1371/journal.pone.0268341 (PMC9352095; doi:10.1371/journal.pone.0268341)
Supplement: S1 Table — a) Low concentration field spikes. b) Medium concentration field spikes. c) High concentration field spikes. (DOCX) [file pone.0268341.s002.docx]

**S1 Table:** Number of QA/QC samples falling within the acceptable 80 to 120% recovery range, including number of samples falling outside that range. a) Low concentration field spikes. b) Medium concentration field spikes. c) High concentration field spikes.

a)

|  | **Number of samples (Percentage of samples)** | | |
| --- | --- | --- | --- |
| **Percent Recovery** | **DEET** | **DHMB** | **DCBA** |
| **<80%** | 7 (30%) | 19 (83%) | 4 (17%) |
| **80-120%** | 4 (17%) | 4 (17%) | 8 (35%) |
| **>120%** | 12 (52%) | 0 (0%) | 11 (48%) |
| **Total** | 23 | 23 | 23 |

b)

|  | **Number of samples (Percentage of samples)** | | |
| --- | --- | --- | --- |
| **Percent Recovery** | **DEET** | **DHMB** | **DCBA** |
| **<80%** | 4 (17%) | 20 (87%) | 0 (0%) |
| **80-120%** | 13 (56%) | 3 (13%) | 16 (70%) |
| **>120%** | 6 (26%) | 0 (0%) | 7 (30%) |
| **Total** | 23 | 23 | 23 |

c)

|  | **Number of samples (Percentage of samples)** | | |
| --- | --- | --- | --- |
| **Percent Recovery** | **DEET** | **DHMB** | **DCBA** |
| **<80%** | 5 (22%) | 20 (87%) | 0 (0%) |
| **80-120%** | 18 (78%) | 3 (13%) | 22 (96%) |
| **>120%** | 0 (0%) | 0 (0%) | 1 (4%) |
| **Total** | 23 | 23 | 23 |
