# Supplementary material for: Development of an observational exposure human biomonitoring study to assess Canadian children’s DEET exposure during protective use
Source: PLoS One. 2022 Aug 4;17(8):e0268341. doi: 10.1371/journal.pone.0268341 (PMC9352095; doi:10.1371/journal.pone.0268341)
Supplement: S2 Table — Limits of detection (LOD) are DEET (0.27 μg/L), DHMB (0.038 μg/L), and DCBA (0.41 μg/L). (DOCX) [file pone.0268341.s003.docx]

**S2 Table:** Concentrations of detected compounds in QA/QC sample field blanks (µg/L). Limits of detection (LOD) are DEET (0.27 µg/L), DHMB (0.038 µg/L), and DCBA (0.41 µg/L).

| **Sample ID** | **DEET** | **DHMB** | **DCBA** |
| --- | --- | --- | --- |
| **Camp 1-1003-1** | <LOD | <LOD | <LOD |
| **Camp 1-1007-1** | 0.32 | <LOD | <LOD |
| **Camp 1-1011-1** | 0.38 | <LOD | <LOD |
| **Camp 1-1066-1** | <LOD | <LOD | <LOD |
| **Camp 1-1070-1** | 0.48 | <LOD | <LOD |
| **Camp 1-1074-1** | <LOD | <LOD | <LOD |
| **Camp 2-0029-1** | <LOD | <LOD | <LOD |
| **Camp 2-0033-1** | <LOD | <LOD | <LOD |
| **Camp 2-0037-1** | <LOD | <LOD | <LOD |
| **Camp 2-0061-1** | <LOD | <LOD | 7.8 |
| **Camp 2-0065-1** | <LOD | <LOD | <LOD |
| **Camp 2-0085-1** | 0.35 | <LOD | <LOD |
| **Camp 2-0090-1** | <LOD | <LOD | <LOD |
| **Camp 2-0093-1** | <LOD | <LOD | <LOD |
| **Camp 2-0109-1** | <LOD | <LOD | <LOD |
| **Camp 2-0113-1** | <LOD | <LOD | <LOD |
| **Camp 2-0117-1** | 1.12 | <LOD | <LOD |
| **Camp 3-2103-1** | 1.14 | <LOD | <LOD |
| **Camp 3-2107-1** | <LOD | <LOD | <LOD |
| **Camp 3-2111-1** | 0.44 | <LOD | <LOD |
| **Camp 3-2203-1** | 0.33 | <LOD | <LOD |
| **Camp 3-2207-1** | 0.35 | <LOD | <LOD |
| **Camp 3-2211-1** | 0.36 | <LOD | <LOD |
