# Supplementary material for: Development of an observational exposure human biomonitoring study to assess Canadian children’s DEET exposure during protective use
Source: PLoS One. 2022 Aug 4;17(8):e0268341. doi: 10.1371/journal.pone.0268341 (PMC9352095; doi:10.1371/journal.pone.0268341)
Supplement: S3 Table — Acceptable percent recovery ranges from 80 to 120% of expected concentrations, as sample concentrations exceed 3 times the limits of detection. (DOCX) [file pone.0268341.s004.docx]

**S3 Table:** Minimum, average, and maximum percent recoveries from all quality assurance and quality control (QA/QC) field samples. Acceptable percent recovery ranges from 80 to 120% of expected concentrations, as sample concentrations exceed 3 times the limits of detection.

|  |  | **All Samples Percent Recovery (%)** | | |
| --- | --- | --- | --- | --- |
| **Concentration** |  | **DEET** | **DHMB** | **DCBA** |
| **Low** | **Minimum** | 57 | 35 | 56 |
| **(n=23)** | **Average** | 163 | 56 | 157 |
|  | **Maximum** | 443 | 115 | 540 |
| **Medium** | **Minimum** | 70 | 41 | 97 |
| **(n=23)** | **Average** | 110 | 59 | 115 |
|  | **Maximum** | 298 | 96 | 143 |
| **High** | **Minimum** | 62 | 43 | 84 |
| **(n=23)** | **Average** | 87 | 59 | 107 |
|  | **Maximum** | 108 | 96 | 122 |
