# Supplementary material for: Development of an observational exposure human biomonitoring study to assess Canadian children’s DEET exposure during protective use
Source: PLoS One. 2022 Aug 4;17(8):e0268341. doi: 10.1371/journal.pone.0268341 (PMC9352095; doi:10.1371/journal.pone.0268341)
Supplement: S4 Table — Camp 1 had a dedicated refrigerator for samples for the duration of the study. Camp 2 and 3 relied on a refrigerated cooler to bring urine samples down to a cool temperature, and then stored urine with ice packs in coolers. a) Low concentration field spikes, anticipated concentrations: DEET 1μg/L; DHMB 0.2μg/L; DCBA 2μg/L. b) Medium concentration field spikes, anticipated concentrations: DEET 8μg/L; DHMB 1.6μg/L; DCBA 16μg/L. c) High concentration field spikes, anticipated concentrations: DEET 128μg/L; DHMB 25.6μg/L; DCBA 256μg/L. (DOCX) [file pone.0268341.s005.docx]

**S4 Table:** QA/QC samples by camp location. Camp 1 had a dedicated refrigerator for samples for the duration of the study. Camp 2 and 3 relied on a refrigerated cooler to bring urine samples down to a cool temperature, and then stored urine with ice packs in coolers. a) Low concentration field spikes, anticipated concentrations: DEET 1µg/L; DHMB 0.2µg/L; DCBA 2µg/L. b) Medium concentration field spikes, anticipated concentrations: DEET 8µg/L; DHMB 1.6µg/L; DCBA 16µg/L. c) High concentration field spikes, anticipated concentrations: DEET 128µg/L; DHMB 25.6µg/L; DCBA 256µg/L.

a)

|  |  | **All Samples Percent Recovery (%)** | | |
| --- | --- | --- | --- | --- |
| **Camp** |  | **DEET** | **DHMB** | **DCBA** |
| **Camp 1** | **Minimum** | 84 | 41 | 74 |
| **(n=6)** | **Average** | 166 | 79 | 123 |
|  | **Maximum** | 280 | 115 | 172 |
| **Camp 2** | **Minimum** | 57 | 56 | 35 |
| **(n=11)** | **Average** | 138 | 198 | 42 |
|  | **Maximum** | 443 | 540 | 55 |
| **Camp 3** | **Minimum** | 103 | 152 | 103 |
| **(n=6)** | **Average** | 115 | 204 | 115 |
|  | **Maximum** | 143 | 283 | 144 |

b)

|  |  | **All Samples Percent Recovery (%)** | | |
| --- | --- | --- | --- | --- |
| **Camp** |  | **DEET** | **DHMB** | **DCBA** |
| **Camp 1** | **Minimum** | 80 | 52 | 99 |
| **(n=6)** | **Average** | 94 | 76 | 106 |
|  | **Maximum** | 108 | 98 | 111 |
| **Camp 2** | **Minimum** | 70 | 41 | 106 |
| **(n=11)** | **Average** | 91 | 49 | 120 |
|  | **Maximum** | 152 | 62 | 143 |
| **Camp 3** | **Minimum** | 111 | 51 | 97 |
| **(n=6)** | **Average** | 159 | 62 | 117 |
|  | **Maximum** | 298 | 70 | 122 |

c)

|  |  | **All Samples Percent Recovery (%)** | | |
| --- | --- | --- | --- | --- |
| **Camp** |  | **DEET** | **DHMB** | **DCBA** |
| **Camp 1** | **Minimum** | 62 | 43 | 84 |
| **(n=6)** | **Average** | 84 | 73 | 98 |
|  | **Maximum** | 95 | 96 | 110 |
| **Camp 2** | **Minimum** | 68 | 43 | 97 |
| **(n=11)** | **Average** | 81 | 50 | 110 |
|  | **Maximum** | 93 | 60 | 123 |
| **Camp 3** | **Minimum** | 81 | 50 | 90 |
| **(n=6)** | **Average** | 99 | 61 | 108 |
|  | **Maximum** | 108 | 67 | 118 |
