# Supplementary material for: Development of an observational exposure human biomonitoring study to assess Canadian children’s DEET exposure during protective use
Source: PLoS One. 2022 Aug 4;17(8):e0268341. doi: 10.1371/journal.pone.0268341 (PMC9352095; doi:10.1371/journal.pone.0268341)

HEALTH CANADA DEET STUDY

Study ID ______

FOR DEET STUDY STAFF ONLY

PARENT/GUARDIAN QUESTIONNAIRE

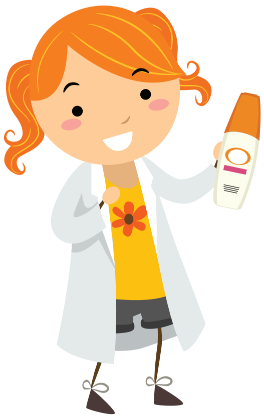

1. Age of your child: __________ years

2. What was the sex of your child at birth?

☐ Female ☐ Male

3. Does your child have any health conditions? ☐ No ☐ Yes (please specify below)*

☐ Asthma
☐ Seizures/Epilepsy
☐ Skin irritations/Eczema/Rosacea
☐ Allergies (specify) ____________________
☐ Kidney problems
☐ Other conditions ____________________

* If you marked yes, and indicated one of the health conditions listed, please contact the study coordinator.

4. Are you aware of Lyme disease?

☐ Yes ☐ No

5. Are you aware of West Nile virus?

☐ Yes ☐ No

6. Highest education level achieved (Parent/Guardian):

☐ Less than secondary school
☐ Secondary school graduation
☐ Some post-secondary
☐ Post-secondary graduation
☐ Prefer not to answer

7. Including all sources, from January to December of last year, what was your annual household income before taxes?

☐ No income
☐ Less than $5,000
☐ $5,000 to $9,999
☐ $10,000 to $14,999
☐ $15,000 to $19,999
☐ $20,000 to $29,999
☐ $30,000 to $39,999
☐ $40,000 to $49,999
☐ $50,000 to $59,999
☐ $60,000 to $79,999
☐ $80,000 to $99,999
☐ $100,000 or more
☐ Prefer not to answer

FOR DEET STUDY STAFF TO REMOVE

CHILD NAME: ______________________________

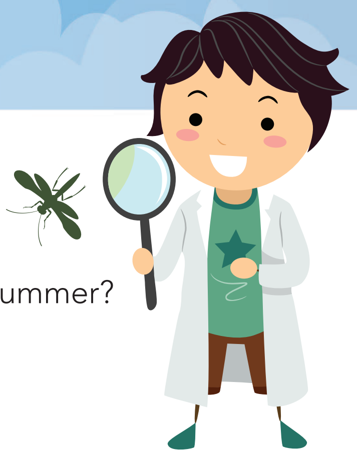

8. How often would you say you and your family uses insect repellent in the summer?

☐ Less than once a month
☐ Less than once a week
☐ 1–2 times per week
☐ 3–4 times per week
☐ 5–6 times per week
☐ Every day

9. What is the name of the brand that you use more regularly, or are currently using, for your child?

☐ Muskol
☐ OFF!
☐ Outbound
☐ Pyramid Trek
☐ Other (specify) ______________
☐ Don't know/don't remember

10. What is the percentage of DEET in your child's bug spray?

☐ 7%
☐ 10%
☐ 15%
☐ 23.5%
☐ 25%
☐ 30%
☐ Don't know
☐ Other (specify) ______________

11. What formulation does your child most regularly use?

☐ Aerosol spray
☐ Pump action spray
☐ Cream
☐ Wipes
☐ Don't know/No preference
☐ Other (specify) ______________

12. Where do you usually buy your child's insect repellent?

☐ Online
☐ In-store
☐ Other (specify) ______________

13. Where do you usually apply insect repellent on your child? (Check all that apply)

☐ Face
☐ Neck and ears
☐ Hair
☐ Arms
☐ Hands
☐ Legs
☐ Feet
☐ Clothing
☐ Other (specify) ______________
☐ All of the above

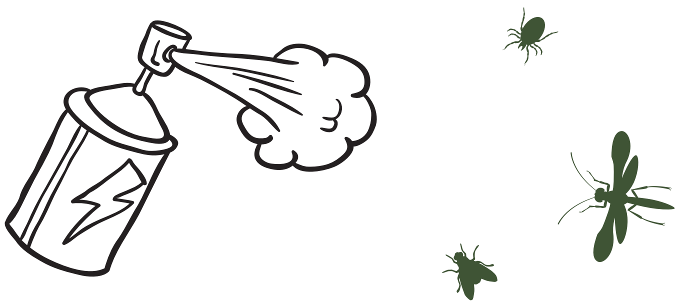

Supplement: S1 File — (PDF) [file pone.0268341.s006.pdf]
