# Supplementary material for: Development of an observational exposure human biomonitoring study to assess Canadian children’s DEET exposure during protective use
Source: PLoS One. 2022 Aug 4;17(8):e0268341. doi: 10.1371/journal.pone.0268341 (PMC9352095; doi:10.1371/journal.pone.0268341)
Supplement: S2 File — (PDF) [file pone.0268341.s007.pdf]

Which bug spray are you using at camp?

BRAND NAME: ______

SIZE: ______ circle one: g or ml

MASS AT BEGINNING: ______ GRAMS

MASS AT END: ______ GRAMS

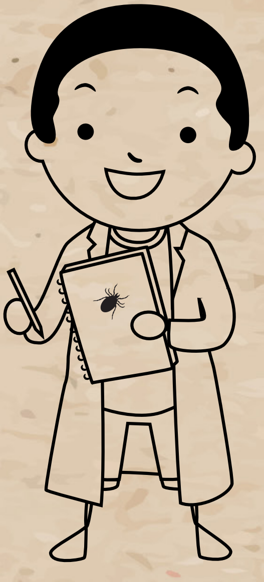

What kind is it?

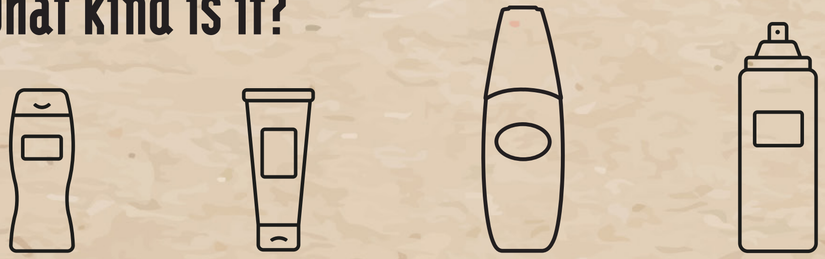

STICK?	LOTION?	PUMP?	SPRAY CAN?
☐	☐	☐	☐

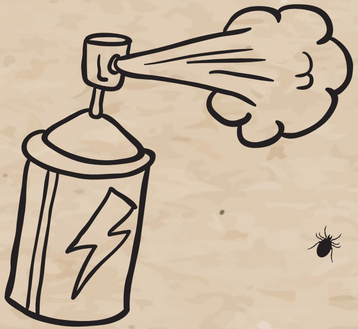

What is in it?

DEET ______ %
AMOUNT

Did you use bug spray? (Check if yes)

☐ YESTERDAY ☐ 2 TO 3 DAYS AGO ☐ LAST WEEK

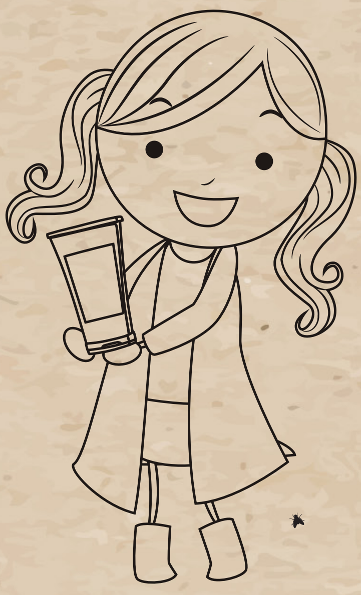

How did you apply your bug spray?

Fill in a new page each time!

What time did you put on bug spray?

______ : ______ AM / PM

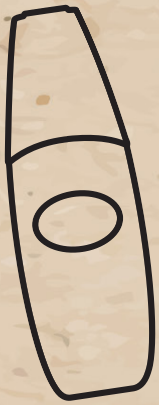

Did you apply any other sprays or lotions or sanitizers? Sunscreen, hand sanitizer, etc.?

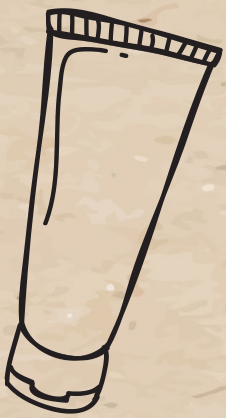

What was it?

[ ]

Where did you apply bug spray? (CHECK IF YES)

- [ ] ARMS
- [ ] HANDS
- [ ] LEGS
- [ ] HAIR
- [ ] FACE
- [ ] CLOTHES
- [ ] NECK AND EARS

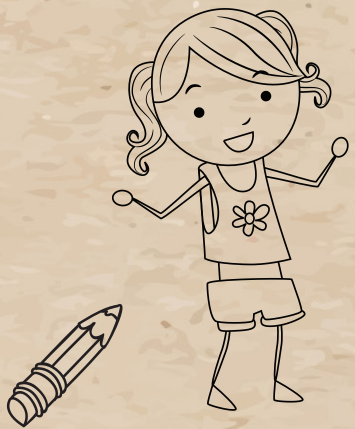

Did you wash your hands after applying the bug spray?

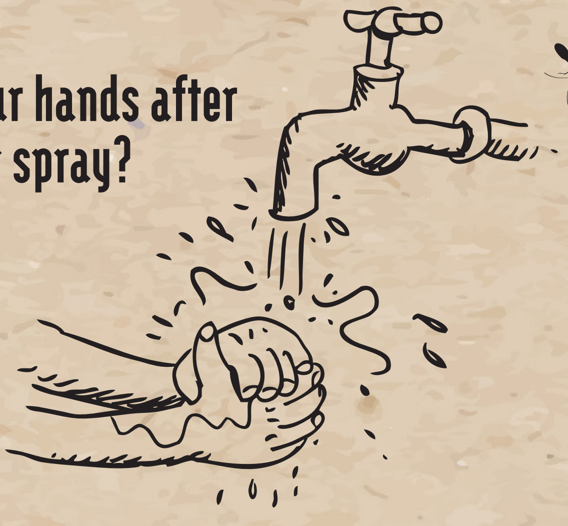

- [ ] YES
- [ ] NO

ALMOST DONE!

A review

GREAT JOB!

☐ Did you pee but not collect it? How many times?

Did you share your bug spray with anyone else?

☐ YES ☐ NO

☐ How many times did you apply bug spray yesterday?

Did you use anyone else's bug spray?

☐ YES ☐ NO

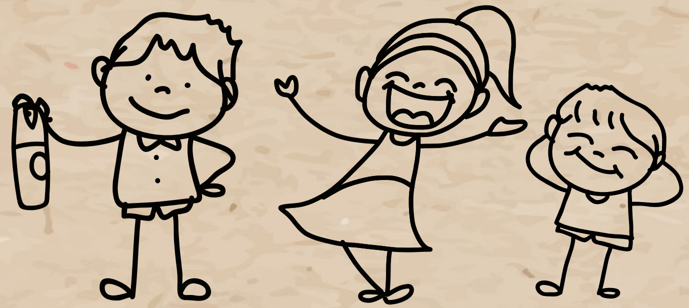

Did you spill your bug spray?

☐ YES ☐ NO

What activities did you do after putting on bug spray?

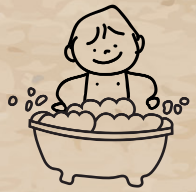

Washed yourself?

☐ Morning ☐ Afternoon ☐ Evening

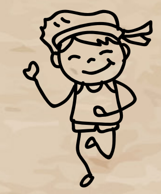

Sweated from exercise?

☐ Morning ☐ Afternoon ☐ Evening

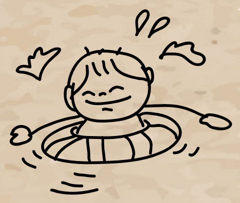

Went swimming?

☐ Morning ☐ Afternoon ☐ Evening

THANK YOU FOR PARTICIPATING!

HAND IN YOUR JOURNAL TO THE DEET STUDY TEAM!
