# Supplementary material for: Development of an observational exposure human biomonitoring study to assess Canadian children’s DEET exposure during protective use
Source: PLoS One. 2022 Aug 4;17(8):e0268341. doi: 10.1371/journal.pone.0268341 (PMC9352095; doi:10.1371/journal.pone.0268341)
Supplement: S3 File — (DOCX) [file pone.0268341.s008.docx]

**Supplementary Material**

**Abbreviations**

B:1-2 – Baseline group: 1-2 days ago

B:LW – Baseline group: Last week

B:N – Baseline group: Never

B:Y – Baseline group : Yesterday

CR - Creatinine

DEET – N,N-diethyl-meta-toluamide

DCBA – 3-diethylcarbamoyl benzoic acid

DHMB – N,N-diethyl-m-(hydroxymethyl)benzamide

ID - Identification

LC-MS/MS – Liquid chromatography – tandem mass spectrometry

LMM – Linear mixed effect model

LOD – Limits of Detection

NHANES – National Health and Nutrition Examination Survey

QA/QC – Quality assurance and quality control

REB – Research Ethics Board

SG – Specific gravity
